# Supplementary material for: Treating symptomatic infections and the co-evolution of virulence and drug resistance
Source: Peer Community J. Author manuscript; Available in PMC 2024 May 4. (PMC7615929; doi:10.24072/pcjournal.38)
Supplement: Appendix [file EMS195833-supplement-Appendix.pdf]

Appendix S1. Supplementary methods

S1.1. Price equation calculations.

Following the Price equation formalism introduced by Day and Proulx (2004), we take the derivative with respect to time of $p_i = I_i / I_T$, we get

$$\frac{\mathrm{d}p_i}{\mathrm{d}t} = \frac{1}{I_T} \left(\frac{\mathrm{d}I_i}{\mathrm{d}t} - \frac{\mathrm{d}I_T}{\mathrm{d}t} \, p_i \right) \tag{S1}$$

From equations 1b and 2a, we get

$$\frac{\mathrm{d}p_i}{\mathrm{d}t} = \left[\left(\beta_i - \overline{\beta}^I \right) \, S - \left(\alpha_i - \overline{\alpha}^I \right) - \left(\gamma_i - \overline{\gamma}^I \right) \right] \, p_i \tag{S2}$$

Let us look at the evolution of the average value of a given trait x. If we assume that the trait of a genotype i does not value with time, we have:

$$\frac{\mathrm{d}\overline{x}}{\mathrm{d}t} = \sum_i x_i \frac{\mathrm{d}p_i}{\mathrm{d}t} \tag{S3}$$

From equation S2, we have:

$$\frac{\mathrm{d}\overline{x}}{\mathrm{d}t} = \sum_i x_i \left[\left(\beta_i - \overline{\beta}^I \right) \, S - \left(\alpha_i - \overline{\alpha}^I \right) - \left(\gamma_i - \overline{\gamma}^I \right) \right] \, p_i \tag{S4}$$

This can also be written as

$$\frac{\mathrm{d}\overline{x}}{\mathrm{d}t} = S \,\mathrm{Cov}\,(\beta, x) - \mathrm{Cov}\,(\alpha, x) - \mathrm{Cov}\,(\gamma, x) \tag{S5}$$

Similarly, we can define the proportion of genotype i in the drug-resistant compartment as $q_i = I_i^R / I_T^R$ and derive it with respect to time to get:

$$\frac{\mathrm{d}q_i}{\mathrm{d}t} = \frac{1}{I_T^R} \left(\frac{\mathrm{d}I_i^R}{\mathrm{d}t} - \frac{\mathrm{d}I_T^R}{\mathrm{d}t} \, q_i \right) \tag{S6a}$$

$$= \rho_i \, \gamma_i \, \frac{I_i}{I_T^R} + \beta_i^R \, S \, q_i - \left(\mu + \nu + \alpha_i^R \right) q_i - \overline{\rho \, \gamma}^I \, \frac{I_T}{I_T^R} \, q_i - \overline{\beta^R}^R \, S \, q_i + \mu + \nu + \overline{\alpha^R}^R \, q_i \tag{S6b}$$

$$= \left(\rho_i \, \gamma_i \, p_i - \overline{\rho \, \gamma}^I \, q_i \right) \frac{I_T}{I_T^R} + \left(\beta_i^R - \overline{\beta^R}^R \right) \, S \, q_i - \left(\alpha_i^R - \overline{\alpha^R}^R \right) \, q_i \tag{S6c}$$

For a given trait y in the drug-resistant compartment, we have

$$\frac{\mathrm{d}\overline{y}}{\mathrm{d}t} = \sum_i y_i \left[\left(\rho_i \, \gamma_i \, p_i - \overline{\rho \, \gamma}^I \, q_i \right) \frac{I_T}{I_T^R} + \left(\beta_i^R - \overline{\beta^R}^R \right) \, S \, q_i - \left(\alpha_i^R - \overline{\alpha^R}^R \right) \, q_i \right] \tag{S7a}$$

$$= \frac{I_T}{I_T^R} \, \sum_i y_i \, \rho_i \, \gamma_i \, p_i - \frac{I_T}{I_T^R} \, \sum_i y_i \, \overline{\rho \, \gamma}^I \, q_i + S \,\mathrm{Cov} \left(\beta^R, y \right) - \mathrm{Cov} \left(\alpha^R, y \right) \tag{S7b}$$

$$\approx \frac{I_T}{I_T^R} \, \left(\overline{y}^I - \overline{y}^R \right) \overline{\rho \, \gamma}^I + S \,\mathrm{Cov}^R \left(\beta^R, y \right) - \mathrm{Cov}^R \left(\alpha^R, y \right) \tag{S7c}$$

The assumption made to reach the last step is that $y_i \, \gamma_i \approx \overline{\gamma}^I \, p_i$.

As before, for the main trait of interest (virulence) and with the same assumptions as before we get:

$$\frac{\mathrm{d}I_T^R}{\mathrm{d}t} = \overline{\rho \, \gamma}^I \, I_T + \overline{\beta^R}^R \, S \, I_T^R - \left(\mu + \nu + \overline{\alpha^R}^R \right) \, I_T^R \tag{S8a}$$

$$\frac{\mathrm{d}\overline{\alpha^R}^R}{\mathrm{d}t} = \frac{I_T}{I_T^R} \, \left(\overline{\alpha^R}^I - \overline{\alpha^R}^R \right) \overline{\rho \, \gamma}^I + S \,\mathrm{Cov}^R \left(\beta^R, \alpha^R \right) - \mathrm{Var}^R \left(\alpha^R \right) \tag{S8b}$$

$$\frac{\mathrm{d}\overline{\beta^R}^R}{\mathrm{d}t} = \frac{I_T}{I_T^R} \, \left(\overline{\beta^R}^I - \overline{\beta^R}^R \right) \overline{\rho \, \gamma}^I + S \,\mathrm{Var}^R \left(\beta^R \right) - \mathrm{Cov}^R \left(\alpha^R, \beta^R \right) \tag{S8c}$$

S1.2. System equilibria.

The detailed equilibria of the main equation system are the following:

(S9a)
$$(\not{S}\,,\,\not{I}\,,\,\not{I}^R) = \left(\frac{\lambda}{\mu}\,,\,0\,,\,0\right)$$

(S9b)
$$(\not{S}\,,\,\not{I}\,,\,\not{I}^R) = \left(\frac{\mu+\nu+\alpha_i+\gamma_i}{\beta_i}\,,\,\frac{\lambda-(\mu+\nu)\not{S}}{\beta_i\,\not{S}\left(\frac{\beta_i^R}{\beta_i}\,\frac{\gamma_i\,\rho_i}{\alpha_i^R+\mu+\nu-\beta_i^R\not{S}}+1\right)}\,,\,\frac{\gamma_i\,\rho_i\,(\lambda-(\mu+\nu)\not{S})}{\not{S}\,(\beta_i^R\,\gamma_i\,\rho_i+\beta_i\,(\alpha^R+\mu+\nu-\beta_i^R\,\not{S}))}\right)$$

(S9c)
$$(\not{S}\,,\,\not{I}\,,\,\not{I}^R) = \left(\frac{\mu+\nu+\alpha_i^R}{\beta_i^R}\,,\,0\,,\,\frac{\lambda}{\mu+\nu+\alpha_i^R}-\frac{\mu+\nu}{\beta_i^R}\right)$$

S1.3. Adaptive dynamics approach.

Deriving the invasion fitness. We now adopt an adaptive dynamics approach to study long term evolution based on the functional relationship between treatment rate and virulence ($\gamma(\alpha)$). For generality, we also initially denote mutation rate as a function of virulence ($\rho(\alpha)$). Finally, we assume a transmission virulence trade-off ($\beta(\alpha)$) otherwise virulence will not be adaptive and always be selected against.

Let us rewrite the ODE system:

(S10a)
$$\frac{dS}{dt} = \lambda - \mu\,S - \beta(\alpha)\,S\,I - \beta^R(\alpha)\,S\,I^R$$

(S10b)
$$\frac{dI}{dt} = \beta(\alpha)\,S\,I - (\mu+\nu+\alpha+\gamma(\alpha))\;I$$

(S10c)
$$\frac{dI^R}{dt} = \rho(\alpha)\,\gamma(\alpha)\,I + \beta^R(\alpha)\,S\,I^R - \left(\mu+\nu+\alpha^R(\alpha)\right)\,I^R$$

Following the next-generation matrix theorem (Diekmann et al., 1990; Driessche and Watmough, 2002; Hurford et al., 2010), the Jacobian matrix of the system (J) can be decomposed into a 'birth' (F) and a 'death' (V) matrix:

(S11a)
$$F = \begin{bmatrix} \beta(\alpha)\,S & 0 \\ \rho(\alpha)\,\gamma(\alpha) & \beta^R(\alpha)\,S \end{bmatrix}$$

(S11b)
$$V = \begin{bmatrix} \mu+\nu+\alpha+\gamma(\alpha) & 0 \\ 0 & \mu+\nu+\alpha^R(\alpha) \end{bmatrix}$$

The eigenvalues of $F.V^{-1}$ are

$$\phi_1 = \frac{\beta^R(\alpha)}{\mu+\nu+\alpha^R(\alpha)}\,S(\alpha) \quad \text{and} \quad \phi_2 = \frac{\beta(\alpha)}{\mu+\nu+\alpha+\gamma(\alpha)}\,S(\alpha)$$

Following the adaptive dynamics framework, we then assume that system S10 is at en epidemiological equilibrium where the parasite persists (see equations S9). This strain is referred to as the 'resident' strain and its trait of interest, virulence, is denoted α_r. We then assume that another strain emerges through mutation that has a slightly different trait value α_m. Key assumptions are that the mutant density is rare compared to that of the resident and that its trait value is close to that of the resident.

Note that if we allow for 'reversions' of drug-resistant infections, the eigenvalues can still be derived but their expression is less clear. We can also see that any link between treatment failure probability and virulence does not matter on the long run.

A perturbation analysis of the system for a rare mutant where the density of susceptible is set by the endemic equilibrium of the resident strain therefore leads to the following eigenvalues

$$\phi_1 = \frac{\beta^R(\alpha_m)}{\mu+\nu+\alpha^R(\alpha_m)}\, S(\alpha_r) \quad \text{and} \quad \phi_2 = \frac{\beta(\alpha_m)}{\mu+\nu+\alpha_m+\gamma(\alpha_m)}\, S(\alpha_r)$$

In this simple model, the ESS corresponds to the strategy that maximises these invasion fitnesses (Dieckmann, 2002). Since the resident trait only affects the density of susceptible hosts, the fitness optimum is an absolute maximum and not a relative maximum.

Evolutionary singular strategies. The first eigenvalue ϕ_1 is identical to that of SI systems studied in details in earlier studies. Depending on the concavity of the transmission-virulence trade-off curve, there can be an ESS with an intermediate level of virulence. More precisely, as shown by M. van Baalen and Sabelis (1995), there is an ESS if there exists a virulence α^* that satisfied the two conditions

(S12a) $$\left.\frac{\mathrm{d}\beta}{\mathrm{d}\alpha_m}\right|_{\alpha_m\to\alpha^*} = \frac{\beta^R(\alpha^*)}{\mu+\nu+\alpha^R(\alpha^*)} \left.\frac{\mathrm{d}\alpha^R}{\mathrm{d}\alpha_m}\right|_{\alpha_m\to\alpha^*}$$

(S12b) $$\left.\frac{\mathrm{d}^2\beta}{\mathrm{d}\alpha_m^2}\right|_{\alpha_m\to\alpha^*} < \frac{\beta^R(\alpha^*)}{\mu+\nu+\alpha^R(\alpha^*)} \left.\frac{\mathrm{d}^2\alpha^R}{\mathrm{d}\alpha_m^2}\right|_{\alpha_m\to\alpha^*}$$

They also show that there is an elegant graphical interpretation to this condition, which is that if there is an ESS, then in α^* the tangent of the parametric curve $(\beta^R(\alpha_m), \mu+\nu+\alpha^R(\alpha_m))$ also passes through $(\mu+\nu, 0)$. Note that treatment rate (γ) has no effect on this ESS.

The second eigenvalue is very similar to the first and, again using the work from M. van Baalen and Sabelis (1995), it can be shown that the second ESS satisfies the following conditions:

(S13a) $$\left.\frac{\mathrm{d}\beta}{\mathrm{d}\alpha_m}\right|_{\alpha_m\to\alpha^*} = \frac{\beta(\alpha^*)}{\mu+\nu+\alpha^*+\gamma(\alpha^*)} \left(1+\left.\frac{\mathrm{d}\gamma}{\mathrm{d}\alpha_m}\right|_{\alpha_m\to\alpha^*}\right)$$

(S13b) $$\left.\frac{\mathrm{d}^2\beta}{\mathrm{d}\alpha_m^2}\right|_{\alpha_m\to\alpha^*} < \frac{\beta(\alpha^*)}{\mu+\nu+\alpha+\gamma(\alpha^*)} \left.\frac{\mathrm{d}^2\gamma}{\mathrm{d}\alpha_m^2}\right|_{\alpha_m\to\alpha^*}$$

Assuming that conditions S13 are satisfied, we can study the effect of variations in treatment rate on the ESS value, which is found by solving equation S13a. This condition can also be written as

(S14) $$f(\alpha^*) = \frac{\mu+\nu+\alpha^*+\gamma(\alpha^*)}{\beta(\alpha^*)} \left.\frac{\mathrm{d}\beta}{\mathrm{d}\alpha_m}\right|_{\alpha_m\to\alpha^*} - \left(1+\left.\frac{\mathrm{d}\gamma}{\mathrm{d}\alpha_m}\right|_{\alpha_m\to\alpha^*}\right) = 0$$

We know from condition S13b that f is a decreasing function of α^*. Therefore, increasing the value of parameter in f will require a larger value of α^* such that $f(\alpha^*) = 0$. For instance, increasing the recovery rate (ν) selects for higher virulences, as expected (Minus van Baalen, 1998). We see that increasing the intensity of the treatment rate favours more virulent strains. However, the faster the treatment rate increases with the virulence, i.e. the larger $\gamma'(\alpha)$, the lower the ESS virulence.

Notice that with the transmission-virulence trade-off function assumed in the main text, we have, for $0 < p < 1$,

(S15) $$f(\alpha^*) = p\,(\mu+\nu+\gamma(\alpha^*)) + \left(p-1-\left.\frac{\mathrm{d}\gamma}{\mathrm{d}\alpha_m}\right|_{\alpha_m\to\alpha^*}\right)\alpha^* = 0$$

Appendix S2. Simulation R code

This appendix contains the R code used for the simulations and generating the figures.

Appendix S3. Supplementary Figures

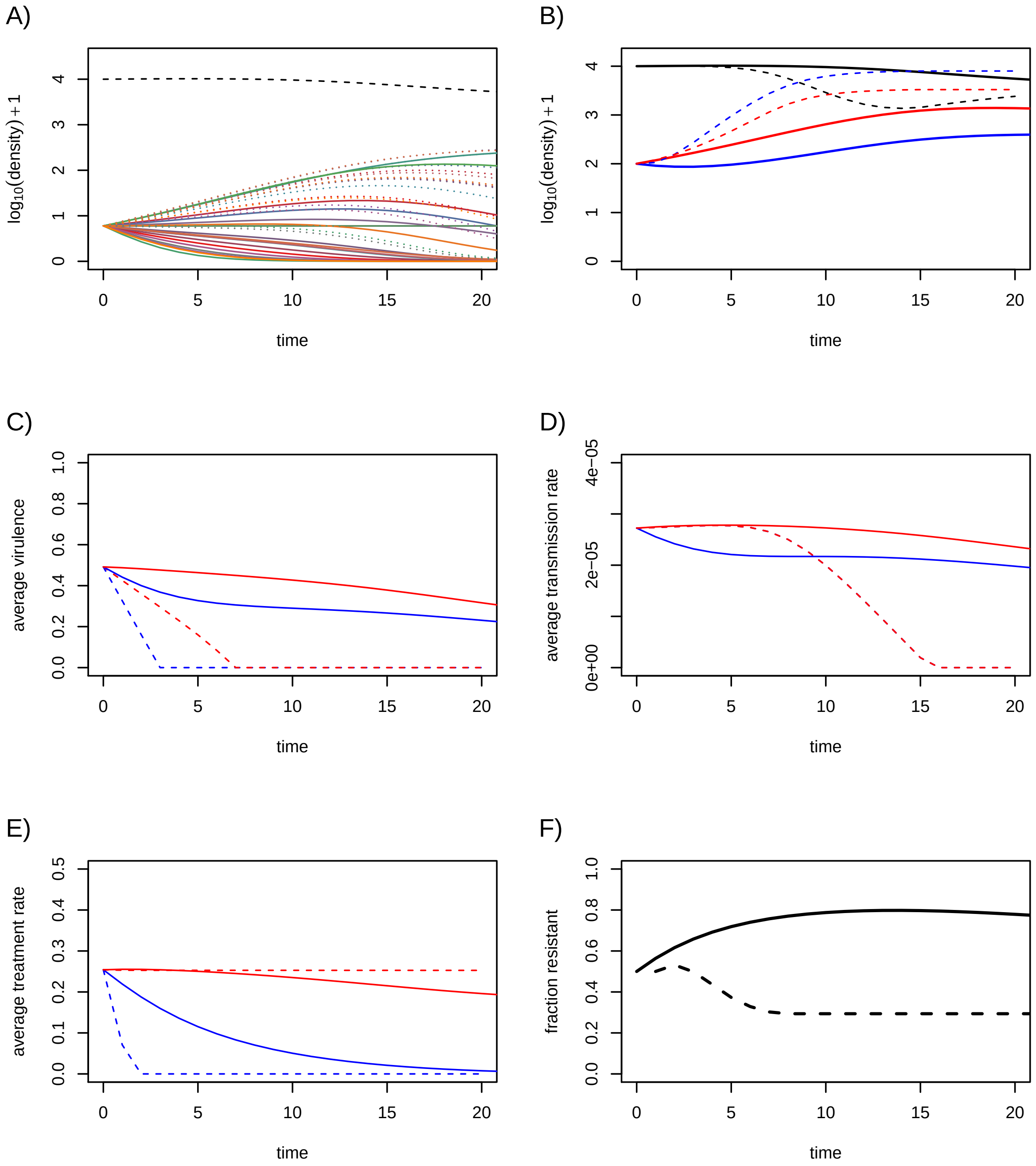

Figure S1 – Short-term dynamics with pre-existing drug resistance. Virulence in the drug-resistant compartment still decreases more slowly than in the drug-sensitive compartment if we assume that the initial density of the two types of infections is equal (therefore that drug resistance is not only generated by treatment failure initially). $I_i^R = 5$ and other parameter values are identical to that in the main text.

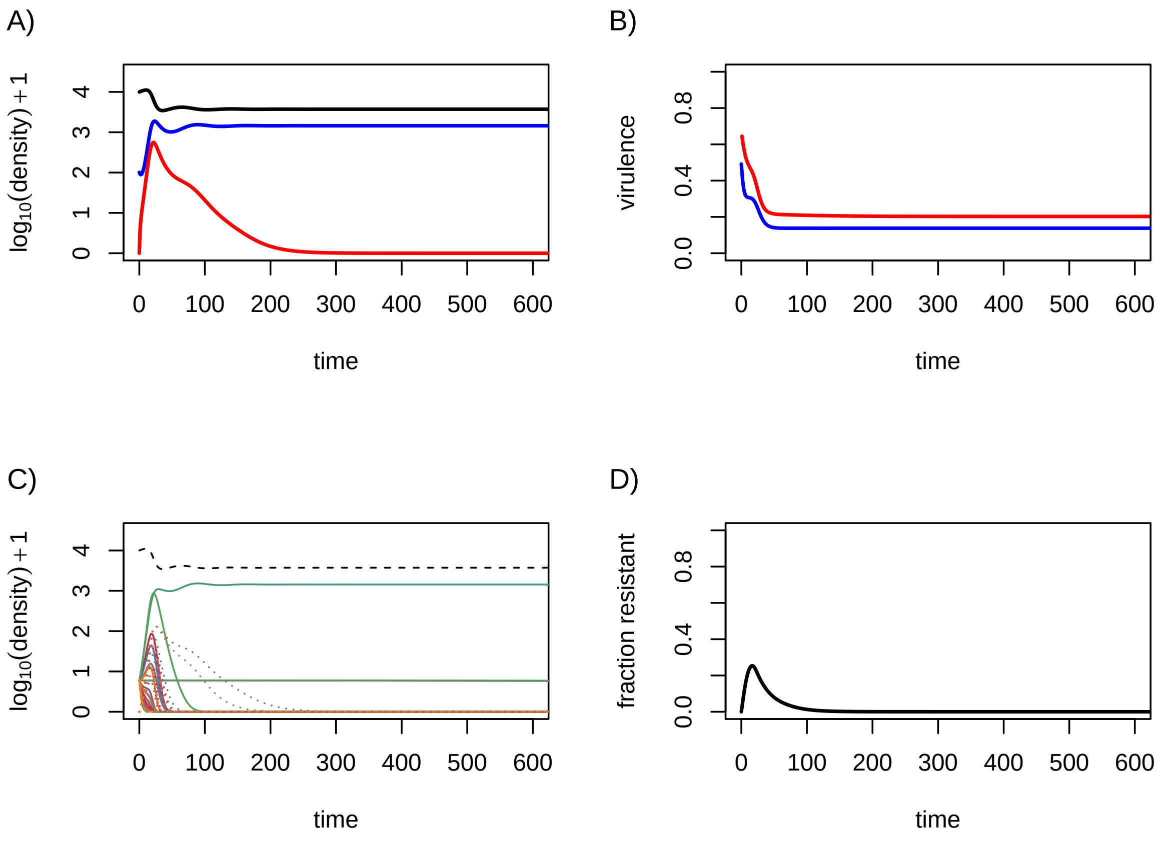

Figure S2 – Long-term evolutionary dynamics. Even with the same parameter values as in the main text, drug-sensitive infections can eventually dominate the system. This is because we only have a limited number of strains in the simulations. Parameter values are the same as in the main text but the $n = 20$ strains are different (although drawn using the same covariance matrix as in the main text).

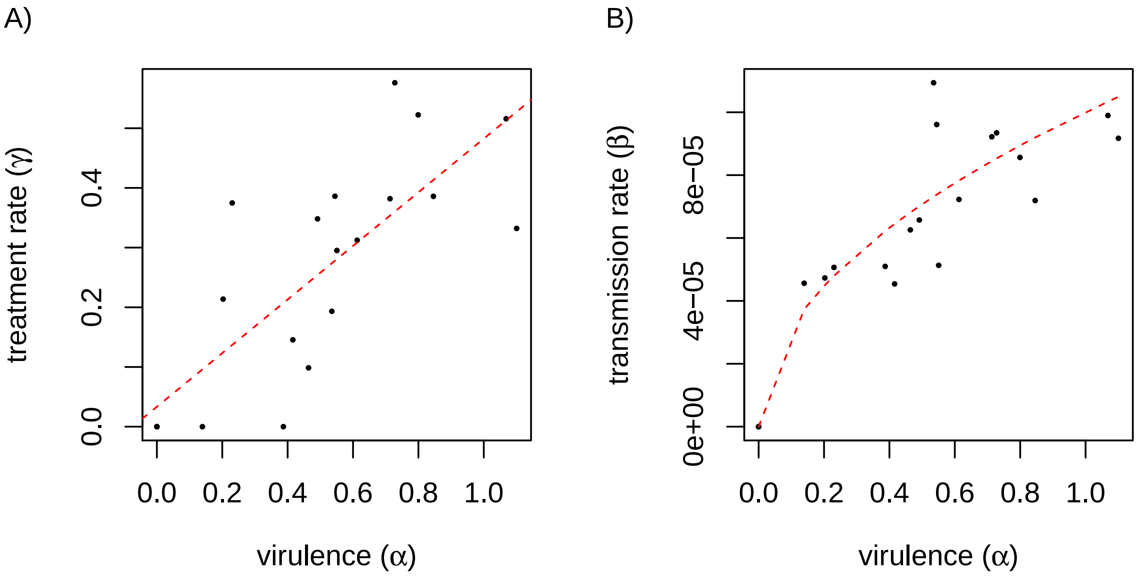

Figure S3 – Trade-off relationships between model parameters. A) Positive covariance between virulence and treatment rate (values are drawn from a multivariate distribution). B) Trade-off relationship assumed between transmission rate and virulence and points obtained when by adding noise to the transmission rates (normal distribution with mean $\beta_0\ \alpha_i^p$ and variance 0.2). Dashed lines show the results of a linear model fit between α and γ in panel A, and the theoretical trade-off relationship from equation 5 for panel B.

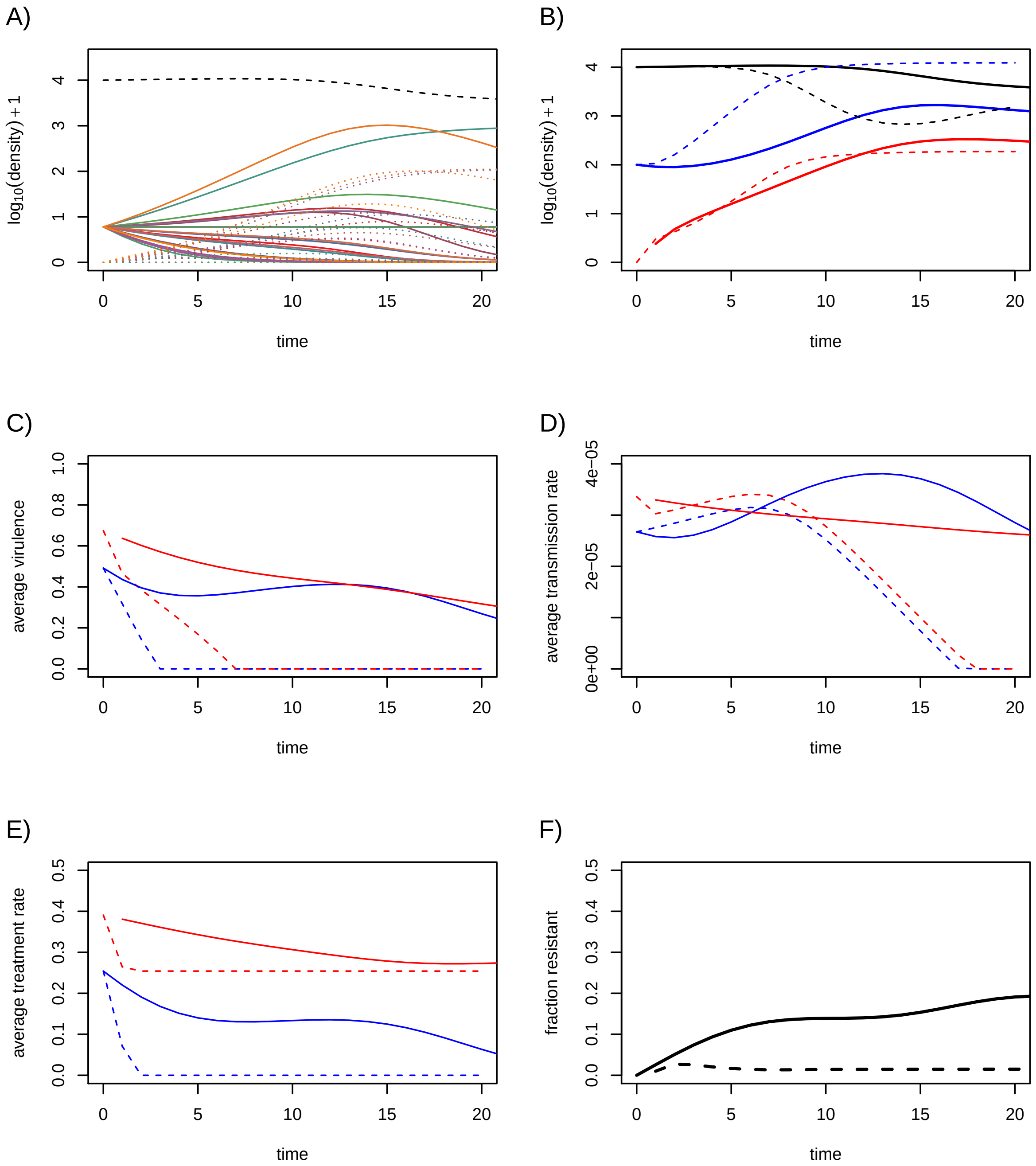

Figure S4 – Short-term evolutionary dynamics assuming noise in transmission rates. Figure captions are identical to that in the main text. Parameter values are identical to that in the main text, except for the noise, which is generated by a Gaussian distribution centred around the transmission value predicted by the trade-off shown in Figure S3B and with relative standard deviation 20%.
